# International, but for whom? Pediatric sepsis guidelines and the limits of global applicability

**DOI:** 10.1371/journal.pgph.0006881

**Published:** 2026-07-10

**Authors:** Johanna Thomson

**Affiliations:** International Paediatric Platform, Médecins sans Frontières (MSF), Paris, France; PLOS: Public Library of Science, UNITED STATES OF AMERICA

Most children who die from sepsis live in low- and middle-income countries (LMICs), yet international pediatric sepsis guidelines are largely derived from evidence generated in high-income intensive care settings. This creates a fundamental tension between global standardization of care and unequal health system capacity. Interventions shown to be effective in well-resourced hospitals may not be feasible, safe, or beneficial in settings with limited monitoring, staffing, and critical care infrastructure.

Recent international pediatric sepsis guidelines, including the 2026 Surviving Sepsis Campaign guidelines, represent a major effort to synthesize evidence and standardize care [1]. However, the generalizability of these recommendations to LMIC settings is uncertain, despite these settings accounting for more than 85% of global pediatric sepsis cases and deaths [2,3]. Many recommendations assume access to intensive care services, including mechanical ventilation, invasive monitoring, vasoactive infusions, reliable laboratory testing, and timely escalation to pediatric intensive care. In many district and referral hospitals, these resources are unavailable or inconsistent. Guidance intended as a standard of care may therefore be difficult to implement safely and consistently in practice.

This reflects a broader imbalance in global pediatric evidence generation. Most clinical trials and observational studies informing sepsis guidelines are conducted in high-income settings with stable infrastructure, specialist staffing, and established safety systems. Although methodologically rigorous, this evidence may not translate reliably to hospitals where staffing ratios are lower, monitoring is intermittent, infusion pumps are scarce, or oxygen supplies are unreliable. The safety and effectiveness of treatment depend partly on the health systems in which it is delivered.

The Fluid Expansion as Supportive Therapy (FEAST) trial remains the clearest example. Conducted in African children with severe infection in hospitals without access to intensive care, the trial showed increased mortality among children receiving fluid bolus therapy compared with maintenance fluids [4]. These findings challenged assumptions derived from high-income practice and demonstrated that treatment effects are context dependent. In settings without mechanical ventilation or intensive monitoring, aggressive fluid resuscitation may carry substantially different risks. The lesson from FEAST is not that evidence generated in high-income settings is invalid, but that evidence cannot be separated from the systems in which care is delivered.

Similar challenges arise in South and Southeast Asia, where severe dengue is a major cause of pediatric shock. Fluid management strategies for dengue differ substantially from those used in bacterial sepsis because of disease-specific pathophysiology, illustrating how recommendations developed for one clinical context may not always be directly transferable to another [5].

Contextual gaps are evident not only in treatment recommendations but also in the evidence base underlying them. Conditions that contribute substantially to pediatric sepsis burden in LMICs, including malaria, dengue, HIV, tuberculosis, sickle cell disease, and severe malnutrition, receive limited emphasis in the evidence underpinning international recommendations [3,6]. This risks reducing the relevance of guidance to the settings where the global burden of disease is greatest.

Yet international guidelines rarely address this uncertainty explicitly. Recommendations are graded according to evidence quality, but seldom according to how resource constraints alter risks and benefits. For clinicians working in resource-limited settings, this creates a practical dilemma: whether to follow recommendations designed for health systems they do not have, or to depart from guidelines without clear evidence-based alternatives.

One solution would be the wider use of resource-stratified recommendations. Tiered guidance for low-, intermediate-, and high-resource settings could improve usability while preserving evidence-based care. In pediatric sepsis, resource-sensitive guidance could include differentiated recommendations for fluid resuscitation, antimicrobial delivery, vasoactive support, and monitoring according to available infrastructure and staffing capacity.

Guideline development processes also remain insufficiently representative of the settings where most pediatric sepsis deaths occur. Although international panels increasingly include contributors from LMICs, leadership and evidence generation remain concentrated in high-income countries.

The format of current guidelines may further limit implementation. Detailed intensive care-focused documents are valuable for specialists but less useful in district hospitals, where most children with sepsis in LMICs are managed by non-specialist clinicians. Simplified algorithms aligned with World Health Organization frameworks could support safer frontline decision making, particularly where staffing and training are limited [7].

These concerns extend beyond sepsis. Similar criticisms have been made of Integrated Management of Childhood Illness protocols, growth standards, and nutritional guidance [8–10]. Across global child health, recommendations often embed implicit assumptions about infrastructure, workforce, and access to care that remain unacknowledged.

Addressing these limitations will require more than minor revision. Major global health funders, including the Bill & Melinda Gates Foundation, Wellcome, the National Institutes of Health, and national research agencies, should prioritize pragmatic trials and implementation research in district and referral hospitals in LMICs. Guideline organizations, including the Surviving Sepsis Campaign and the World Health Organization, should develop formal resource-stratified recommendations and ensure leadership from clinicians and researchers working in high-burden settings. Guidelines should explicitly acknowledge resource constraints and provide clear pathways for adaptation across different levels of health system capacity.

The aim of global pediatric guidelines should not be rigid uniformity, but improved outcomes across diverse clinical settings. More than a decade after FEAST and decades after similar concerns were raised across global child health programs, resource constraints remain inadequately incorporated into many international recommendations. Continued reliance on guidance that assumes unavailable infrastructure risks perpetuating inequities in care rather than reducing them.

## References

[1] Weiss SL, Peters MJ, Oczkowski SJW, Belle-Cote E, Buysse C, Choong KLM, et al. Surviving Sepsis Campaign International Guidelines for the Management of Sepsis and Septic Shock in Children. Pediatr Crit Care Med. 2026. doi: 10.1097/PCC.0000000000003927 41869844

[2] Tan B, Wong JJ-M, Sultana R, Koh JCJW, Jit M, Mok YH, et al. Global Case-Fatality Rates in Pediatric Severe Sepsis and Septic Shock: A Systematic Review and Meta-analysis. JAMA Pediatr. 2019;173(4):352–62. doi: 10.1001/jamapediatrics.2018.4839 30742207 PMC6450287

[3] Rudd KE, Johnson SC, Agesa KM, Shackelford KA, Tsoi D, Kievlan DR, et al. Global, regional, and national sepsis incidence and mortality, 1990–2017: analysis for the Global Burden of Disease Study. Lancet. 2020;395(10219):200–11. 31954465 10.1016/S0140-6736(19)32989-7 PMC6970225

[4] Maitland K, Kiguli S, Opoka RO, Engoru C, Olupot-Olupot P, Akech SO, et al. Mortality after fluid bolus in African children with severe infection. N Engl J Med. 2011;364(26):2483–95. doi: 10.1056/NEJMoa1101549 21615299

[5] World Health Organization. Comprehensive guidelines for prevention and control of dengue and dengue haemorrhagic fever. New Delhi: WHO. 2011.

[6] Fleischmann-Struzek C, Goldfarb DM, Schlattmann P, Schlapbach LJ, Reinhart K, Kissoon N. The global burden of paediatric and neonatal sepsis: a systematic review. Lancet Respir Med. 2018;6(3):223–30. doi: 10.1016/S2213-2600(18)30063-8 29508706

[7] World Health Organization. Pocket book of hospital care for children: guidelines for the management of common childhood illnesses. 2nd ed. Geneva: WHO. 2013. 24006557

[8] Bryce J, Victora CG, Habicht J-P, Black RE, Scherpbier RW, MCE-IMCI Technical Advisors. Programmatic pathways to child survival: results of a multi-country evaluation of Integrated Management of Childhood Illness. Health Policy Plan. 2005;20 Suppl 1:i5–17. doi: 10.1093/heapol/czi055 16306070

[9] de Onis M, Onyango AW, Borghi E, Siyam A, Nishida C, Siekmann J. Development of a WHO growth reference for school-aged children and adolescents. Bull World Health Organ. 2007;85(9):660–7. doi: 10.2471/blt.07.043497 18026621 PMC2636412

[10] Kerac M, McGrath M, Connell N, Kompala C, Moore WH, Bailey J, et al. “Severe malnutrition”: thinking deeply, communicating simply. BMJ Glob Health. 2020;5(11):e003023. doi: 10.1136/bmjgh-2020-003023 33208313 PMC7677332

